# A New Toolbox to Label Zinc-MTF1 Responsive Neuronal Populations Unravels Cellular Congruence between MTF1 Responses and T-type Calcium Channelopathies in an Experimental Model of Epilepsy

**DOI:** 10.1007/s12035-025-05433-z

**Published:** 2025-12-13

**Authors:** Annachiara Meconi, Katharina Schmied, Aniella Bak, Julika Pitsch, Henner Koch, Susanne Schoch, Albert J. Becker, Karen M. J. van Loo

**Affiliations:** 1 Institute of Cellular Neurosciences II (IZN II), Medical Faculty, University of Bonn, Bonn, Germany; 2 Department of Epileptology, Neurology, RWTH Aachen University, Aachen, Germany; 3 Department of Epileptology, University Hospital Bonn, Bonn, Germany; 4 Department of Neurosurgery, RWTH Aachen University, Aachen, Germany

**Keywords:** Metal-regulatory transcription factor-1, Zinc, Transcriptional reporter unit, T-type calcium channel Ca_V_3.2, Epileptogenesis

## Abstract

Temporal lobe epilepsy (TLE) is one of the most common forms of epilepsy and usually manifests after serious brain insults, including traumatic brain injury, stroke, and status epilepticus (SE). Previously, we identified a new epilepsy-associated transcriptional control mechanism that relies on the zinc-dependent transcriptional activator metal-regulatory transcription factor-1 (MTF1). MTF1 is one of the key transcription factors involved in intracellular metal ion balance. Upon excessive increase in the concentration of zinc in the cytoplasm, zinc can bind to MTF1, inducing its translocation to the nucleus to activate the expression of metal-response element (MRE)-containing genes, including metallothioneins and the T-type calcium channel Ca_V_3.2. To date, in-depth analyses of MTF1 downstream signaling cascades have been limited by the lack of tools to visualize Zn^2+^/MTF1-responsive neuronal activation *in vivo*. To overcome this challenge, we developed a transcriptional reporter unit that enables genetic labeling of Zn^2+^/MTF1-expressing cells *in vitro*, *ex vivo* and *in vivo*. Applying this new toolbox in a mouse model for epileptogenesis revealed a clear overlap between Zn^2+^/MTF1-induced cellular localization and activity of a promoter-driven reporter for Ca_V_3.2, supporting the calcium channel as a direct target of the Zn^2+^/MTF1-cascade. This innovative toolbox provides a powerful resource for studying zinc-induced MTF1 activation in both physiological and pathological processes, including epileptogenesis.

**Supplementary Information:**

The online version contains supplementary material available at 10.1007/s12035-025-05433-z.

## Introduction

One of the main mechanisms responsible for altered protein expression, cellular distribution, and functionality in the context of disease is the differential expression of transcription factors [1, 2]. Previously, we described a novel transcriptional mechanism in the process of epileptogenesis based on zinc-dependent activation of the transcription factor Metal-regulatory Transcription Factor 1 (MTF1) [3]. MTF1 is evolutionarily well conserved [4] and can mediate the transcription of numerous genes, including metallothionein genes, the zinc transporter ZnT1, and the T-type calcium channel Ca_V_3.2 [3, 5–9]. Under physiological conditions, MTF1 localizes to the cytoplasm and nucleus [10], and upon activation of MTF1 by exposure to zinc and cadmium or by cellular stressors like hypoxia or oxidative stress, MTF1 accumulates in the nucleus [11, 12]. Within the nucleus, MTF1 can bind to specific DNA motifs known as Metal Responsive Element (MRE) sequences, which typically conform to the consensus sequence 5’-TGCRCNC-3’. These elements are generally located in the promoter regions of target genes, where MTF1 binding facilitates transcriptional activation [13, 14].

MTF1 plays a central role in heavy metal homeostasis and detoxification by regulating the expression of genes that directly affect intracellular heavy metal levels or availability [15–17]. Zinc ions (Zn^2+^) are, after iron, the most abundant metal ions in cells and are required by all organisms for proper cellular metabolism and structural scaffolding of many proteins [18, 19]. A deficiency or excess of Zn^2+^ can cause various diseases, including immune dysfunction, growth retardation, and sensory and motor neuropathies [19–21]. Zn^2+^ also plays an important role during periods of intense neuronal activity, such as status epilepticus (SE), when Zn^2+^ is released from glutamatergic terminals [22].

Building on our observation that Zn^2+^, although capable of acutely and reversibly blocking T-type calcium channels [23], can also augment the T-type low-voltage activated Ca^2+^ current (*I*_CaT_) in CA1 pyramidal neurons [24], we further characterized this dual effect as a novel mechanism of neuronal plasticity termed the ‘Zn^2+^-MTF1-Ca_v_3.2 cascade of epileptogenesis’ [3]. Interestingly, interference with this cascade attenuates the emergence of spontaneous seizures in the chronic phase [3]. In addition, the same cascade has been implicated in social stress-induced anxiety-like behavior in the distal region of the ventral CA3 hippocampus [9]. These results indicate that, in addition to metal homeostasis and detoxification, MTF1 may play a pivotal role in converting a normal hippocampal network to hyperexcitability.

Despite significant advancements in our understanding of MTF1’s role in neuronal plasticity and metal homeostasis, a critical gap remains in the ability to visualize and label Zn^2+^/MTF1-activated cells *in vivo*. Existing detection systems for intracellular Zn^2+^ primarily rely on chemical indicators or fluorescent probes (e.g. FluoZin-3, Zinquin, or the genetically encoded Zn^2+^ sensor Zinpyr-1) that measure overall Zn^2+^ levels within the cells [25]. While these tools are valuable for assessing Zn^2+^ concentrations, they lack specificity regarding which cells are activated by Zn^2+^/MTF1 signaling. Also, the use of immunohistochemistry applying antibodies against proteins involved in Zn^2+^ transport or binding represents a suitable strategy. While it provides spatial information about protein expression, it does not directly measure Zn^2+^/MTF1-bound, and thus, functional MTF1 expression levels. These aspects limit our ability to fully elucidate the functional implications of the Zn^2+^/MTF1 signaling pathway.

Further investigation of Zn^2+^/MTF1-activated cells in physiological and pathological contexts requires visualization techniques that specifically reflect their activation state. Although tools have been developed to detect intracellular Zn^2+^ in cultured neurons [26, 27], genetic tools for labeling Zn^2+^/MTF1-activated cellular populations *in vivo* are lacking. In this study, we developed a transcriptional reporter unit that enables the labeling of Zn^2+^/MTF1-expressing cells *in vitro*, *ex vivo,* and *in vivo* for in-depth analysis of Zn^2+^/MTF1-responsive neuronal activation, and used this new toolbox in a rodent model of epileptogenesis.

## Materials and Methods

### Plasmids

#### MRE Reporter Constructs

**Fig. 1:**
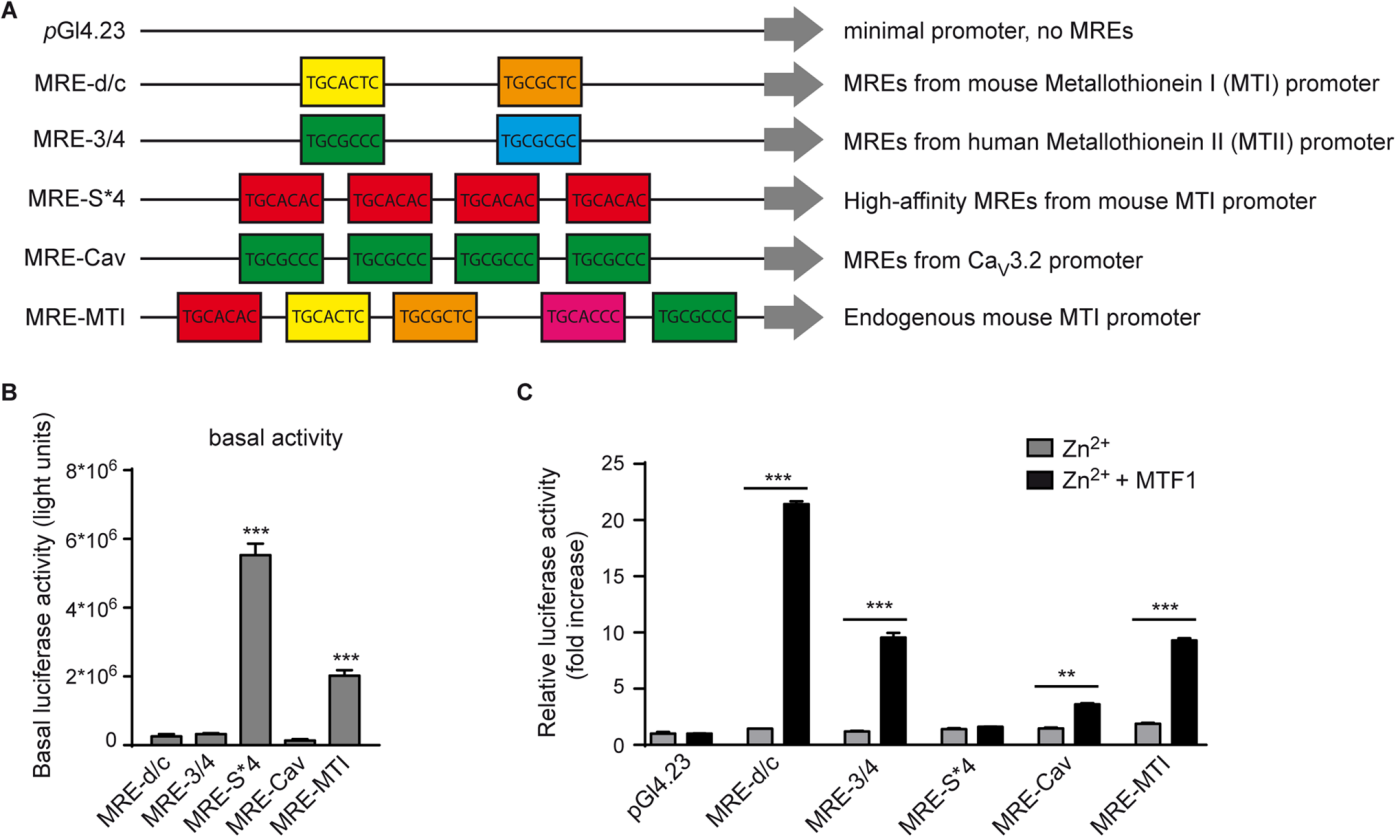
Increases in Zn^2+^/MTF1 activate several MRE-containing transcriptional units *in vitro.* (**A**) Schematic overview of the transcriptional units. Potential metal responsive elements (MREs) are indicated by colored boxes, with each color representing a different MRE consensus sequence. The *p*Gl4.23 transcriptional unit only harbors a minimal promoter without any MREs. (**B**) Basal luciferase activity of the five transcriptional units, normalized to the basal expression of the MRE-lacking *p*Gl4.23 transcriptional unit in NG108-15 cells. A strong basal luciferase activity was observed for MRE-S*4 and MRE-MTI (One-way ANOVA: *P* < 0.001, F(5,12) = 188.8, Tukey’s multiple comparisons test, ***P ≤ 0.001; *N* = 3, *n* = 3). (**C**) Luciferase activity of Zn^2+^-challenged NG108-15 cells transfected with the transcriptional units with and without MTF1. A significant increase in luciferase activity was observed for MRE-d/c (21.4-fold), MRE-3/4 (9.5-fold), MRE-Cav (3.6-fold) and MRE-MTI (9.3-fold) after Zn.^2+^/MTF1 application. No increase was observed for MRE-S*4 (One-way ANOVA: *P* < 0.001, F(11,22) = 1196, Tukey’s multiple comparisons test, **P ≤ 0.01, ***P ≤ 0.001; *N* = 3 independent experiments, *n* = 3 replicates per condition)

Five different MRE sequences were selected based on putative MTF1 binding: MRE-d/c, MRE-3/4, MRE-S*4, MRE-Cav, and MRE-MTI (Fig. 1A). Three of these (MRE-d/c, MRE-3/4, and MRE-S*4) have been previously described and were selected based on demonstrated MTF1 binding in vitro [7, 28, 29]. The MRE-Cav construct was derived from the *Cacna1h* promoter, which encodes the Ca_V_3.2 T-type calcium channel. This construct is based on a promoter fragment previously validated to exhibit Zn^2+^-induced MTF1 binding in an animal model of temporal lobe epilepsy [3]. Specifically, MRE-Cav contains four tandem repeats of MREs that are present within the upstream regulatory region of the endogenous *Cacna1h* gene. These MREs are also partially shared with the sequences used in the MRE3/4 and MRE-MTI constructs (Fig. 1A). Importantly, the MRE-Cav construct does not encompass the full-length *Cacna1h* promoter, and thus does not include additional MTF1-binding elements that may contribute to the full transcriptional responsiveness *in vivo*. MRE-MTI was developed based on the endogenous mouse *MTI* promoter, which contains five binding sites for MTF1 [30].

First, all five MRE sequences were cloned into the *p*Gl4.23 reporter vector (Promega: #9PIE841), a luciferase reporter plasmid containing a minimal promoter (minP) upstream of the luciferase gene. The MRE sequences were inserted into the multiple cloning site (MCS) located upstream of the minimal promoter such that any increase in luciferase expression in our assays reflects activation by the inserted MRE element.

MRE-d/c, MRE-3/4, MRE-S*4 and MRE-Cav were cloned into *p*Gl4.23 by oligonucleotide annealing and ligation. The oligonucleotide sequences used were: MRE-d/c: forward (FW): 5’-gatctatcgataattctctgcactccgcccgaaaagtgcgctcgga-3’ and reverse (RV): 5’-agcttccgagcgcacttttcgggcggagtgcagagaattatcgata-3’. MRE-3/4: FW: 5’- gatctatcgataattcggtgcgcccggcccagtgcgcgcggccga-3’ and RV: 5’-agcttcggccgcgcgcactgggccgggcgcaccgaattatcgata-3’. MRE-S*4: FW: 5’-gatctatcgatgctctgcacacggccgctctgcacacggccgctctgcacacggccgctctgcacacggcca-3’ and RV: 5’-agcttggccgtgtgcagagcggccgtgtgcagagcggccgtgtgcagagcggccgtgtgcagagcatcgata-3’ primers. MRE-Ca_v_: FW: 5’-gatctatcgatgctctgcgcccccgcgctctgcgcccccgcgctctgcgcccccgcgctctgcgcccccgca-3’ and RV: 5’-agcttgcgggcgcagagcgcgggggcgcagagcgcgggggcgcagagcgcgggggcgcagagcatcgata-3’. Oligos were annealed by mixing equimolar amounts in annealing buffer (100 mM Tris pH 7.5, 1M NaCl, 10 mM EDTA), heating at 94 °C for 10 min, and slowly cooling to room temperature. Annealing generated a BglII restriction site at the 5’ end and a HindIII site at the 3’ end. The annealed oligos were phosphorylated using polynucleotide kinase (10 U/µl) and ligated into a BglII/HindIII-digested *p*Gl4.23 vector.

For the MRE-MTI transcriptional unit, the MTI sequence was amplified by PCR using mouse genomic DNA as template with the primer set FW: (BglII) 5’- gcgagatctatcgatgtcccgctgtgcacactg-3’ and RV: (HindIII) 5’- gcgaagcttcgagtccgggcgcaaag-3’. The PCR product was purified, digested with BglII and HindIII, and ligated into the BglII/HindIII-digested *p*Gl4.23 vector.

To construct the adeno-associated virus (AAV) plasmids expressing the Venus fluorescent protein under control of the MRE elements, we replaced the Ca_v_3.2 promoter in the pAAV-Ca_v_3.2-Venus vector [3] with each MRE transcriptional unit from the* p*Gl4.23 constructs (described above). Each MRE-*p*Gl4.23 construct was digested with MluI and HindIII, and the MRE fragments were gel-purified and ligated into the MluI/HindIII-digested pAAV-Ca_v_3.2-Venus backbone.

To create the pAAV-MRE-Luciferase plasmids, the Venus cassette in each pAAV-MREs-Venus construct was removed by NcoI and BsrGI digestion, and replaced with the firefly luciferase sequence. Luciferase was amplified from *p*Gl4.23 using the primer set FW: 5’-cagcttacaaccatgatggaagatgccaaaaacattaagaagg-3’ and RV: 5’-atctcttacttgtacttacacggcgatcttgccgc-3’.

The pAAV-MTI-*Lac*Z plasmid was made by exchanging the Venus sequence from the NcoI/BsrGI-digested pAAV-MTI-Venus construct with the *Lac*Z sequence amplified from pAAV-CMV-*LacZ* (Addgene #105,531) using FW: 5’-cagcttacaaccatgatgaccatgattacggattcactgg-3’ and RV 5’-atctcttacttgtacttatttttgacaccagaccaactggt-3’. The pAAV-MTI-IRFP^713^ was cloned by replacing the hSynapsin promoter from the MluI/XbaI digested pAAV-hSyn-iRFP^713^ vector with the MRE-MTI transcriptional unit, amplified by PCR from pGl4.23-MRE-MTI-luciferase using FW: 5’-ctgcggccgcacgcgggtacctgagctcgctagcctcg-3’ and RV: 5’- ccatggtggctctagggtggctttaccaacagtaccg-3’.

pAAV-MTI-mRuby3 was made by replacing the Venus sequence of pAAV-MTI-Venus with the mRuby3 sequence. The mRuby3 sequence was amplified by PCR from pAAV-CMV-mRuby3 with FW: 5’-cagcttacaaccatggtgtctaagggcga-3’ and RV: 5’- atctcttacttgtacttacttgtacagctcgtccatgcc-3’ and cloned into the NcoI/BsrGI-digested pAAV-MTI-Venus backbone using the In-Fusion® HD Cloning kit (Takara Bio Europe/Clontech).

Due to the limited AAV packaging capacity and the large size of the *Lac*Z protein, pAAV-Ca_v_3.2-*Lac*Z was created by replacing the full-length Ca_v_3.2 promoter [31] with a deletion fragment (Ca_v_3.2–1020), which retains full transcriptional activity [31]. The fragment was inserted into a BamHI/SalI-digest pAAV-MCS-*Lac*Z vector. The Ca_v_3.2–1020 deletion fragment was generated by PCR using rat genomic DNA as a template with primer FW: 5’-gcgacgcgtcagtgaagggaaggggcggcgc-3′ and RV: 5’-gcggtcgacgtggcggagggcagcac-3’.

#### Other Plasmids

The pAAV-hSyn-MTF1-IRES-Venus, pAAV-MTF1ΔC-IRES-Venus (dominant negative variant) and the pAAV-hSyn-RL-TK have been described previously [3]. To generate pAAV-hSyn-tdTomato and pAAV-hSyn-GFP, the tdTomato and GFP sequences were PCR amplified and ligated into the BamHI/BgIII-digested pAAV-hSyn-Venus vector [3]. The pAAV-hSyn-MTF1-2A-SBFP2 construct was assembled by inserting the MTF1 coding sequence (without stop codon) and the 2A-SBFP2 sequence (from pKan-CMV-2A-SBFP2; kind gift from D. Wachten, Bonn University) into the HindIII/XbaI-digested pAAV-hSyn-MCS vector using In-Fusion cloning (Takara Bio Europe/Clontech). The MTF sequence was amplified using FW: 5’-ccggggatcctctagaatgggggaacacagtccagac-3’ and RV: 5’-tgtttcagggtggcagctgcagg-3’ primers and the 2A-SBFP2 sequence using FW: 5’-gccacccgtgaaacagactttgaattttgaccttctcaag-3’ and RV: 5’- tgctcgaggcaagctttcacttgtacagctcgtccatgc-3’ primers. Primers were designed such that the MTF1 reverse primer overlapped with the forward primer for 2A-SBFP2 to allow fusion during the In Fusion reaction.

All AAV-cloning procedures were performed in Stellar competent cells (Takara Bio Europe/Clontech) to minimize recombination. Plasmid sequences were verified by sequencing analysis.

### Cell Culture and Virus Production

NG108-15 cells (ATCC® HB-12317TM) were maintained at 37 °C and 5% CO_2_ in Dulbecco's Modified Eagle's Medium (DMEM) supplemented with 10% (v/v) heat inactivated fetal calf serum (FCS) (Gibco®), 100 units ml^−1^ penicillin/streptomycin, 2 mM glutamine, and 1 × HAT (sodium hypoxanthine, aminopterin and thymidine; Invitrogen). HEK293-AAV cells (Stratagene, La Jolla, CA) were maintained in high glucose DMEM supplemented with 10% FCS (Gibco®), 100 units ml^−1^ penicillin/streptomycin, and 2 mM glutamine and incubated at 37 °C and 5% CO_2_.

Primary hippocampal neurons were prepared from C57B/l-6N mice. Pregnant mice were sacrificed under deep isoflurane anesthesia (Piramal Critical Care), and the uterus containing embryos (embryonic days 14–18) was removed. Hippocampi and cortex were isolated from embryos, and tissue was washed three times with cold Ca^2+^/Mg^2+^ free Hanks' Balanced Salt Solution HBBS (Gibco®) before being digested with 200 µl 2.5% trypsin (10X) (Gibco®) for 20 min at 37°C. After three washing steps with HBSS, the medium was aspirated and 200 µl 1mg/ml DNase-I (Roche) together with 800 µl 1X basal medium eagle (BME) (Gibco®) was added. Tissue was mechanically dissociated by trituration using a 1 ml plastic tip. Neurons were counted, seeded in a 24-wells plate coated with Poly-D-Lysine (Sigma) and kept at 37 °C, 5%CO_2_ in BME supplemented with 0.5% glucose (Sigma-Aldrich), 10% FCS (Gibco®), 2% B-27 (Life Technologies), and 0.5 mM L-glutamine (Gibco®). Recombinant AAV1/2 genomes were generated by large-scale triple CaPO_4_ transfection of HEK293-AAV cells as previously described [3].

### Luciferase Assay

*Luciferase assay on NG108-15 cells*. NG108-15 cells were transfected using lipofectamine®2000 (Invitrogen, USA) in 48-well plates (60′000 cells/well). Per well, 0.05 µg of pAAV-MRE-Luciferase plasmids (MRE-d/c, MRE-3/4, MRE-S*4, MRE-Cav and MRE-MTI), 0.0125 µg of *Renilla* control vector (pRL-TK; Promega), and 0.025 µg pAAV-hSyn-MTF1 were mixed with 0.5 µl lipofectamine and 25 µl OPTI-MEM medium (Invitrogen, USA) and added to the appropriate wells after 20 min RT incubation. Cells were grown in Opti-MEM™ medium (Gibco®) for 16 h. The serum-free medium was then replaced by normal NG108-15 medium (see above). Forty-eight hours after transfection, the cells were incubated in normal NG108-15 medium or in normal medium with 50 mM KCl (Carl-Roth®) and 200 µM ZnCl_2_ (Sigma-Aldrich). Four hours after stimulation, cells were returned to normal medium and lysed. Luciferase activity was measured using the Dual Luciferase Reporter Assay System (Promega). Briefly, cells were washed with Phosphate Buffer Saline (PBS) and incubated for 15 min with the appropriate amount of Passive Lysis Buffer (PLB). Subsequently, 20 µl/well of cell lysate was transferred in a 96 well Lumitrac™ plate (Greiner Bio-one, Germany) and combined with 100 µl Luciferase Assay Reagent II (LAR II) and 100 µl Stop & Glo®. *Renilla* and firefly luciferase activities were measured using the Glomax Luminometer (Promega). Results are given as firefly/*Renilla* relative light units.

*Luciferase assay on hippocampal neurons*. Hippocampal neurons (24 wells plate, 40 ‘000 neurons/well) were cotransduced on DIV7 with 5 µl rAAV-MREs-Luciferase, 5 µl rAAV-TK-Renilla and with 5 µl rAAV-hSyn-MTF1-IRES-Venus viral suspension. On DIV14, neurons were incubated with 1 µM ZnCl_2_ for 1 h. Luciferase activities were determined after 4 h using the Dual Luciferase Report Assay System as described previously, and the results are expressed as firefly/*Renilla* relative light units.

### Time-Lapse Imaging

Hippocampal neurons (24 well plates, 40′000 neurons/well) were transduced on DIV1 with 5 µl rAAV-MTI-Venus viral suspension. Experimental wells were transduced with 5 µl rAAV-MTF1-2A-SBFP2 viral suspension on DIV7 and treated for 1 h with 1 µM ZnCl_2_ on DIV 14. Control wells were treated in the same way without ZnCl_2_ but incubated with normal medium. Neurons were monitored and photographed under a fluorescence microscope (Axio Observer, A.1, Zeiss) to observe changes in fluorescence intensity before Zn^2+^ stimulation. Pictures (20X) were taken under the same conditions using a Jenoptik ProgRes MF Cool camera. Two hours after Zn^2+^ stimulation, changes in fluorescence intensity were examined using time-lapse imaging (Nikon Eclipse Ti microscope) for 15 h. Photographs (40X) were taken using a DS-Qi2 camera (Nikon).

### Organotypic Brain Slice Cultures

Mouse organotypic brain slice cultures (OBSCs) were prepared from male and female mice (C57Bl6/N) on postnatal days 4–8 as described before [32, 33]. In brief, brains were isolated and cut in 350 μm thick coronal cortical slices using a vibratome (Leica VT1200S). Slices were cultured in 6-well plates with uncoated 30 mm Millicell-CM tissue culture inserts with 0.4 µm pores in Minimum Essential Media (Thermo Fisher Scientific) containing 20% horse serum, 1 mM L-glutamine, 0.00125% ascorbic acid, 0.001 mg/ml insulin, 1 mM CaCl_2_, 2 mM MgSO_4_, 13 mM D-glucose, and 1% penicillin–streptomycin. rAAVs were applied to the slices at DIV0-1. The medium was changed every 48–72 h.

### Multi-electrode Array Recordings

Multi-electrode array (MEA) recordings on transduced OBSCs were performed using 16 × 16 electrodes MEA chips (256MEA30/8iR-ITO-pr, electrode size 30 μm, 200 μm spacing, Multi Channel Systems MCS GmbH). Slices were weight down by a meshed harp and continuously perfused with oxygenated aCSF (95% O_2_, 5% CO_2_) at a temperature of 30 ± 1 °C. Signals were sampled at 10 kHz after an equilibration period of 5 min using Multi Channel Experimenter software (Multi Channel Systems MCS GmbH). Spontaneous activity was recorded in three 5-min sessions. Recordings were analyzed using custom-written scripts in MatLab (MathWorks, RRID:SCR_001622), as described previously in detail [33]. Briefly, the data were filtered with a 50 Hz low-pass filter, and three network-driven local field potentials (LFPs) were analyzed per recording. The mean voltage values of the positive and negative amplitudes of the three LFPs per recording were then calculated and compared between the cortex and hippocampus.

### Staining of OBSCs

At the end of the experiment, slices were fixed overnight in 4% paraformaldehyde at 4 °C and subsequently washed in PBS (3 × 15 min). Next, the slices were incubated in 15% sucrose solution for 90 min, followed by a 30% sucrose solution overnight. After washing the slices in PBS (3 × 15 min) and blocking for 1 h in 10% normal goat serum in PBS-T, they were incubated for two days at 4 °C with anti-NeuN (1:1000, MAB377, Millipore) and anti-GFP antibodies (1:800, Ab290, Abcam, Cambridge UK). After washing with PBS, the slices were incubated for 24 h at 4 °C with Alexa Fluor Plus 488 and Alexa Fluor Plus 555 secondary antibodies (1:750, Thermo Fisher Scientific). Finally, the slices were incubated for one minute in DAPI (D9542, Merck), washed with PBS, and mounted using Fluoromount-G (Thermo Fisher Scientific). Fluorescence activity was measured using the REVOLUTION upright and inverted microscope system (Echo) and the LSM710 confocal microscope system (Zeiss).

### Stereotactic Viral Vector Injection

Adult male mice (~ 56 days, > 20 gr) were obtained from Charles River (C57Bl/6-N) and housed under a 12 h light/dark cycle in a temperature (22 ±  2 °C) and humidity (55 ± 10%) controlled environment with food and water ad libitum. Animals were allowed 1 week of adaptation to the animal facility before surgery. Mice were anesthetized with 0,05 mg kg ^−^1 fentanyl (B. Braun, Germany), 5,0 mg kg ^−^1 midazolam (Rotexmedica GmH, Germany) and 0,5 mg kg ^−^1 medetomidine (Dorbene, Parsippany, NJ), intraperitoneally (i.p.). Thirty minutes before anesthesia, mice received a subcutaneous (s.c.) injection of 5 mg kg^−1^ ketoprofen (Rifen, Vetoquinol). Intracerebral AAV injections in the left and right CA1 hippocampal regions were performed stereotactically after drilling a hole through the skull at the coordinates (in mm): −2 AP, −1.5/1.5 ML, and −1.5 DV relative to the bregma. Next, 1 μl of viral suspension containing ~ 10^8^ transducing units was injected through a 10 μl syringe at a rate of 200 nl min^−1^ using a microprocessor-controlled mini-pump (World Precision Instruments). After injection, the needle was left in place for 5 min before removal, the incision was closed, and mice were allowed to recover on a heating plate. Mice were monitored twice per day and received a s.c. analgesic (Ketoprofen, Rifen, Vetoquinol) injection for 3 days postoperatively. All animal procedures were planned and performed to minimize pain and suffering, and to reduce the number of animals used in accordance with European, national, and institutional guidelines (guidelines of the European Parliament and of the Council on the protection of animals used for scientific purposes, European Directive (2010/63/EU), and the ARRIVE guidelines). The study protocol was approved by the Landesamt für Natur, Umwelt und Verbraucherschutz (LANUV) of North Rhine-Westphalia, Germany (approval number 81–02.04.2019.A248).

### Pilocarpine-induced SE

To induce SE, C57Bl/6-N mice (> 20 g) were injected with a single dose of the muscarinic agonist pilocarpine hydrochloride (335 mg kg^−1^, s.c., Sigma). To avoid peripheral muscarinic effects, scopolamine methyl nitrate (1 mg kg^−1^, s.c., Sigma) was administered 20 min before pilocarpine injection. Forty minutes after SE onset, the mice were injected once with diazepam (4 mg kg^−1^, s.c., Ratiopharm). SE was defined as sustained continuous convulsions with loss of postural [34]*.* Control mice were administered scopolamine methyl nitrate and diazepam, but 0.9% NaCl (Fresenius) instead of pilocarpine. After SE, all animals were fed a 5% glucose solution (Fresenius) and soaked rodent food, and checked twice per day.

### LacZ Staining

Adult male C57Bl/6-N mice were injected as described above, either with rAAV-MTI-*Lac*Z or a combination of rAAV-MTI-*Lac*Z and rAAV-hSyn-MTF1-2A-SBFP2 (rAAV-MTF1) or a combination of rAAV-MTI-*Lac*Z and rAAV-hSyn-GFP (rAAV-GFP). For the Ca_v_3.2-*Lac*Z experiments, adult male mice C57Bl/6-N were injected with rAAV-Ca_v_3.2-*Lac*Z. Two weeks after injection, mice were sham- or pilocarpine-SE treated. Three days after SE, mice were decapitated under deep isoflurane anesthesia. Brains were rapidly removed, coronal slices (400 µm) were made on a vibratome (Thermo Fisher Scientific), and β-Galactosidase staining was performed according to the manufacturer’s protocol (Sigma Aldrich, #GALS_1 kit). Slices were recut in 50 µm, mounted in mowiol mounting medium (Roth, Germany), and acquired using the Zeiss AxioScan Z1 slide scanner provided by the Microscope Core Facility of the University of Bonn. Mice injected with an overexpression of rAAV-GFP and rAAV-MTF1 were sacrificed 2 weeks after injection, and *Lac*Z staining was performed as described above.

### Near-infrared *in vivo* Imaging

Mice were injected with either rAAV-MTI-iRFP or rAAV-MTI-iRFP combined with rAAV-hSyn-MTF1-2A-SBFP2 particles (rAAV-MTF1) or rAAV-MTI-iRFP combined with rAAV-hSyn-GFP (rAAV-GFP). Three weeks after injection, the mice were anesthetized and basal iRFP values were measured through the skull. Three days after basal iRFP measurements, mice underwent pilocarpine-induced SE or sham treatment. iRFP values were determined again 2, 10, and 28 days after pilocarpine-induced SE (time points were selected based on the results described in [35]). The iRFP intensity signal of animals injected with an overexpression of rAAV-GFP and rAAV-MTF1 was measured 2 weeks after injection.

Near-infrared imaging was performed using a Pearl® Impulse Small Animal Imaging System (Li-COR Biosciences GmbH, Bad Homburg, Germany). The iRFP signal was determined using a highly sensitive charged coupled device (CCD) camera. The excitation and emission wavelengths were fixed at 690 and 710 nm, respectively. Pictures were analyzed using the Pearl® Impulse Image Studio Software v3.1 (Li-COR Biosciences GmbH, Bad Homburg, Germany). Fluorescent signals were normalized to background levels and quantified by placing two round regions of interest (ROI; Fig. 4E) above the hippocampal region. Fluorescent signals are presented as arbitrary units (a.u.).

### Brain Slice Imaging

Adult male C57Bl/6-N mice were injected with either a combination of rAAV-MTI-mRuby3 and rAAV-Ca_v_3.2-Venus or with and without rAAV-hSyn-MTF1-2A-SBFP2. Three weeks after injection, the animals received sham or pilocarpine-SE treatment. Mice were perfused 3 days after SE with 4% paraformaldehyde (PFA). Brains were removed and left overnight in 4% PFA. Coronal slices (25–50 µm) were made using a vibratome (Thermo Fisher Scientific), mounted, and images (20X) were taken with a Nikon Eclipse Ti microscope.

### Experimental Design and Statistical Analysis

Statistical analyses were performed using GraphPad Prism 6.05 software. Sample sizes for each experiment were chosen based on prior experience with similar assays and to ensure adequate statistical power to detect biologically meaningful differences. Where possible, power calculations were performed a priori using effect sizes observed in preliminary experiments (aiming for power of 0.8 and α 0.05) using GPower3.1.

All results are given as mean ± standard error of the mean (SEM). Statistical significance was assessed using appropriate tests based on experimental design and data distribution: two-tailed Student’s *t*-tests were used for comparisons between two groups, one-way ANOVA followed by Tukey’s multiple comparison tests for comparisons involving multiple groups with one independent variable, and two-way ANOVA followed by Sidak´s multiple comparison tests for experiments involving two independent variables. Normality of data distribution and homogeneity of variances were checked to justify the use of parametric tests. *P*-values are indicated as * P ≤ 0.05, ** P ≤ 0.01, *** P ≤ 0.001. All tests were two-sided, and statistical methods and exact sample sizes for each experiment are detailed in the figure legends or Results section.

### Data Availability

All data generated or analysed during this study are included in this article.

## Results

### Identification of Zn^2+^-sensitive MTF1 Transcriptional Units *in vitro*

To investigate the function of Zn^2+^/MTF1-activated cells, it is essential to visualize cells activated by MTF1 in a Zn^2+^-dependent manner. To develop a transcriptional reporter unit capable of genetically labeling such cells, we first designed five different transcriptional constructs containing multiple potential MTF1 binding sites (metal-responsive elements; MREs), designated MRE-d/c, MRE-3/4, MRE-S*4, MRE-Cav, and MRE-MTI (for a detailed description of the MREs, see Materials and Methods section, Fig. 1A). Each of these constructs was cloned upstream of a firefly luciferase reporter gene to allow quantitative assessment of transcriptional activity.

The five MRE-based reporter constructs were tested for their Zn^2+^-inducible MTF1 sensitivity in neuronal NG108-15 cells using luciferase assays. Under basal conditions, strong luciferase activity was observed for MRE-S*4, and to a lesser extent for MRE-MTI, while MRE-d/c, MRE-3/4, and MRE-Cav showed minimal baseline activity (Fig. 1B).

To assess Zn^2+^-responsiveness, we elevated intracellular Zn^2+^ concentrations ([Zn^2+^]_i_), using 200 µM Zn^2+^ and 50 mM KCl for 4 h, a paradigm previously shown to activate the Zn^2+^/MTF1-cascade in neuronal NG108-15 cells [3]. Although some constructs exhibited minor changes in luciferase activity under these conditions, only MRE-MTI showed a significant Zn^2+^-induced increase (Suppl Fig. 1). Upon MTF1 overexpression combined with Zn^2+^ treatment, significant luciferase induction was observed for MRE-d/c, MRE-3/4, MRE-Cav, and MRE-MTI, while MRE-S*4 showed no further increase in activity (Fig. 1C).

### The Mouse Metallothionein I Promoter Functions as a Zn^*2*+^-Sensitive MTF1 Reporter Unit in Hippocampal Neurons

**Fig. 2:**
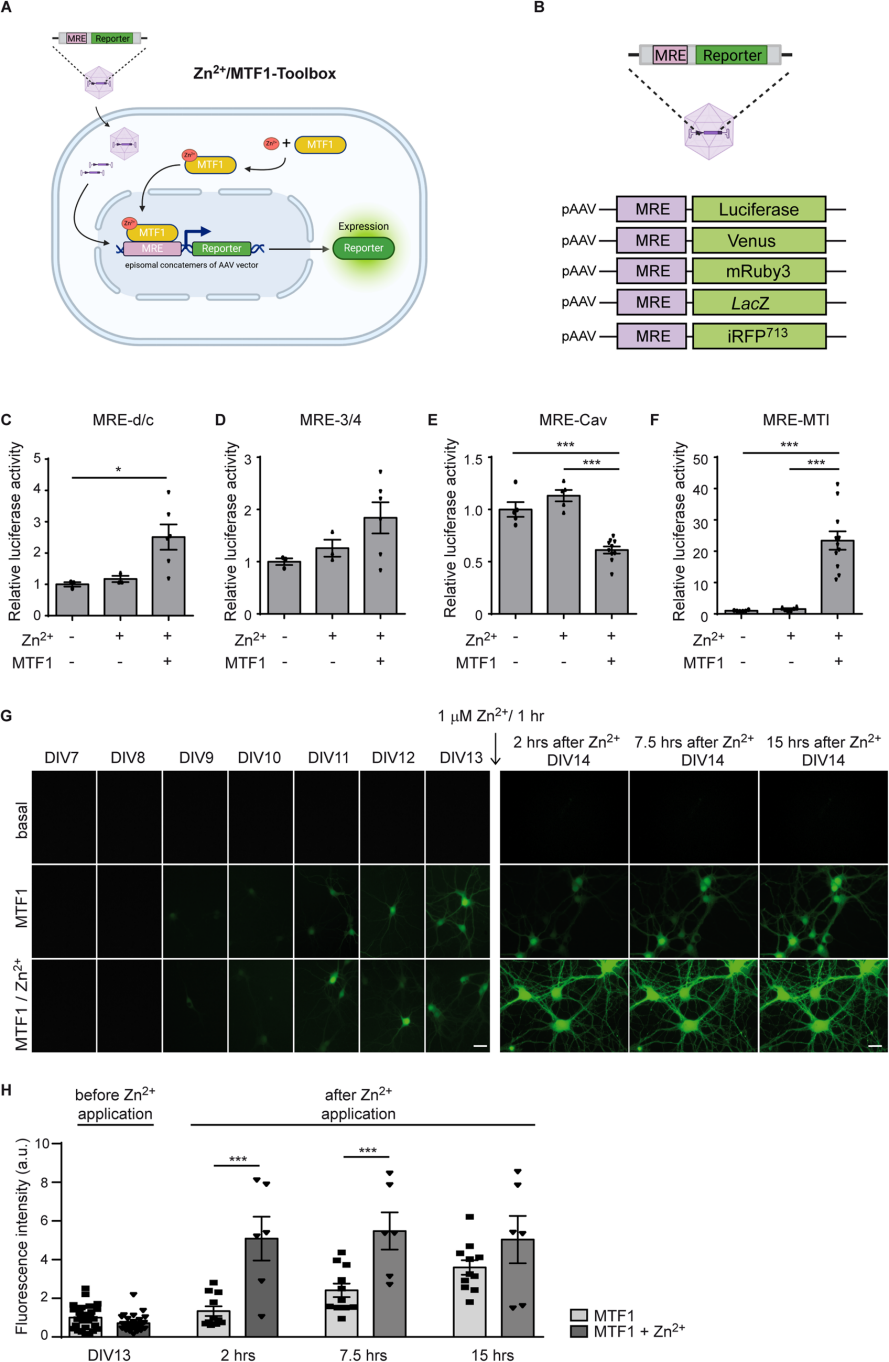
The mouse MTI transcriptional unit functions as a Zn^2+^-sensitive MTF1 reporter unit in mouse hippocampal neurons. (**A**, **B**) Schematic representation of the (**A**) Zn^2+^/MTF1-toolbox (MRE: metal responsive element, MTF1: metal regulatory transcription factor 1) and (B) of the viral MRE constructs used in this study. Created with BioRender.com. (**C-F**) Luciferase activity of the MRE-d/c (**C**), MRE-3/4 (**D**), MRE-Cav (**E**) and MRE-MTI (**F**) transcriptional units, normalized against the luciferase activity of the *p*Gl4.23 transcriptional unit. Mouse hippocampal neurons were transduced at days in vitro (DIV) 7 with the transcriptional units, with and without MTF1. At DIV14, cells were Zn^2+^-challenged (1 µM, 1 h) and luciferase activity was measured 4 h after Zn^2+^-challenge. A significant increase after Zn^2+^/MTF1-challenge was observed for MRE-d/c (One-way ANOVA: *P* = 0.027, F(2,9) = 5.54, Tukey’s multiple comparisons test, *P ≤ 0.05, *N* = 3, *n* ≥ 3) and MRE-MTI (One-way ANOVA: *P* < 0.001, F(2,20) = 29.23, Tukey’s multiple comparisons test, ***P ≤ 0.001; *N* = 3, *n* ≥ 3). (**G**) Fluorescence intensity of mouse primary hippocampal neurons transduced with rAAV-MTI-Venus (DIV1), rAAV-hSyn-MTF1-2A-SBFP2 (DIV7), and stimulated with Zn^2+^ solution (DIV14; 1 µM Zn^2+^ for 1 h). Fluorescence intensity was measured daily from DIV7 until DIV13. After Zn^2+^ incubation, fluorescence intensity was measured 2, 7.5 and 15 h after washing out the Zn^2+^ solution. Under basal conditions (rAAV-MTI-Venus) no fluorescence signal was detected longitudinally. After co-transduction with rAAV-hSyn-MTF1-2A-SBFP2 (MTF1 panel), fluorescence starts around DIV9. Subsequent Zn^2+^ incubation (MTF1/Zn^2+^ panel) results in a strong fluorescence intensity already 2 h after incubation. Scale bars 25 µm. (**H**) Quantification of the fluorescence intensity shown in E. Data were normalized against the fluorescence intensity before Zn.^2+^-application (DIV13). (One-way ANOVA: *P* < 0.001, F(7,97) = 24.92, Tukey’s multiple comparisons test, ***P ≤ 0.001; *N* = 3 independent experiments, *n* ≥ 3 replicates per condition)

Next, we tested the four Zn^2+^/MTF1-sensitive transcriptional units that showed increased reporter activity following Zn^2+^/MTF1 stimulation (MRE-d/c, MRE-3/4, MRE-Cav and MRE-MTI) in primary hippocampal neurons. Primary hippocampal neurons (DIV 7) were transduced with rAAVs expressing luciferase under the control of these transcriptional units (Fig. 2A, B). While application of Zn^2+^ alone (1 µM Zn^2+^, 1 h) did not result in significant changes in luciferase activity, overexpression of MTF (via rAAV-hSyn-MTF1-IRES-Venus application at DIV7), combined with Zn^2+^ stimulation, led to a significant increase in luciferase activity for the MRE-d/c and MRE-MTI transcriptional units (Fig. 2C-F). Since the MRE-MTI transcriptional unit showed a markedly stronger activation in response to Zn^2+^/MTF1-stimulation (MRE-MTI: 23.4-fold; *p* ≤ 0.001) compared to MRE-d/c (2.5-fold; *p* = 0.0441), and also exhibited a strong activation in NG108-15 cells, we chose to focus on the MRE-MTI transcriptional unit in our subsequent experiments.

To prove unequivocally that the MTI transcriptional unit can be activated by MTF1 in a Zn^2+^-sensitive manner, primary hippocampal neurons were transduced (DIV 1) with rAAVs harboring the Venus protein under the control of the MRE-MTI transcriptional unit (rAAV-MTI-Venus, Fig. 2B), followed by MTF1 overexpression (transduction of rAAV-hSyn-MTF1-2A-SBFP2 at DIV 7). As compatible with the presence of physiologically low level free Zn^2+^ within the neurons, a slightly increased abundance of fluorescence intensity was observed one day before Zn^2+^ application (DIV 13) for neurons with MTF1 overexpression (Fig. 2G, middle and lower panels). This gradual increase likely reflects the accumulation of virally expressed MTF1, which is expected to begin around DIV10-DIV11 following transduction at DIV7. Subsequent incubation of the cells in Zn^2+^ solution (DIV 14; 1 µM Zn^2+^, 1 h) resulted in a robust increase in fluorescence intensity measured at 2 and 7.5 h after Zn^2+^ application, which no further increased at 15 h after Zn^2+^ application (Fig. 2G, H). Taken together, these findings confirm that MRE-MTI is a transcriptional unit that can be used to label Zn^2+^/MTF1-activated cells *in vitro*.

### Activation of the MTI Transcriptional Unit by Zn^2+^/MTF1 in *ex vivo* Organotypic Brain Slice Cultures

**Fig. 3:**
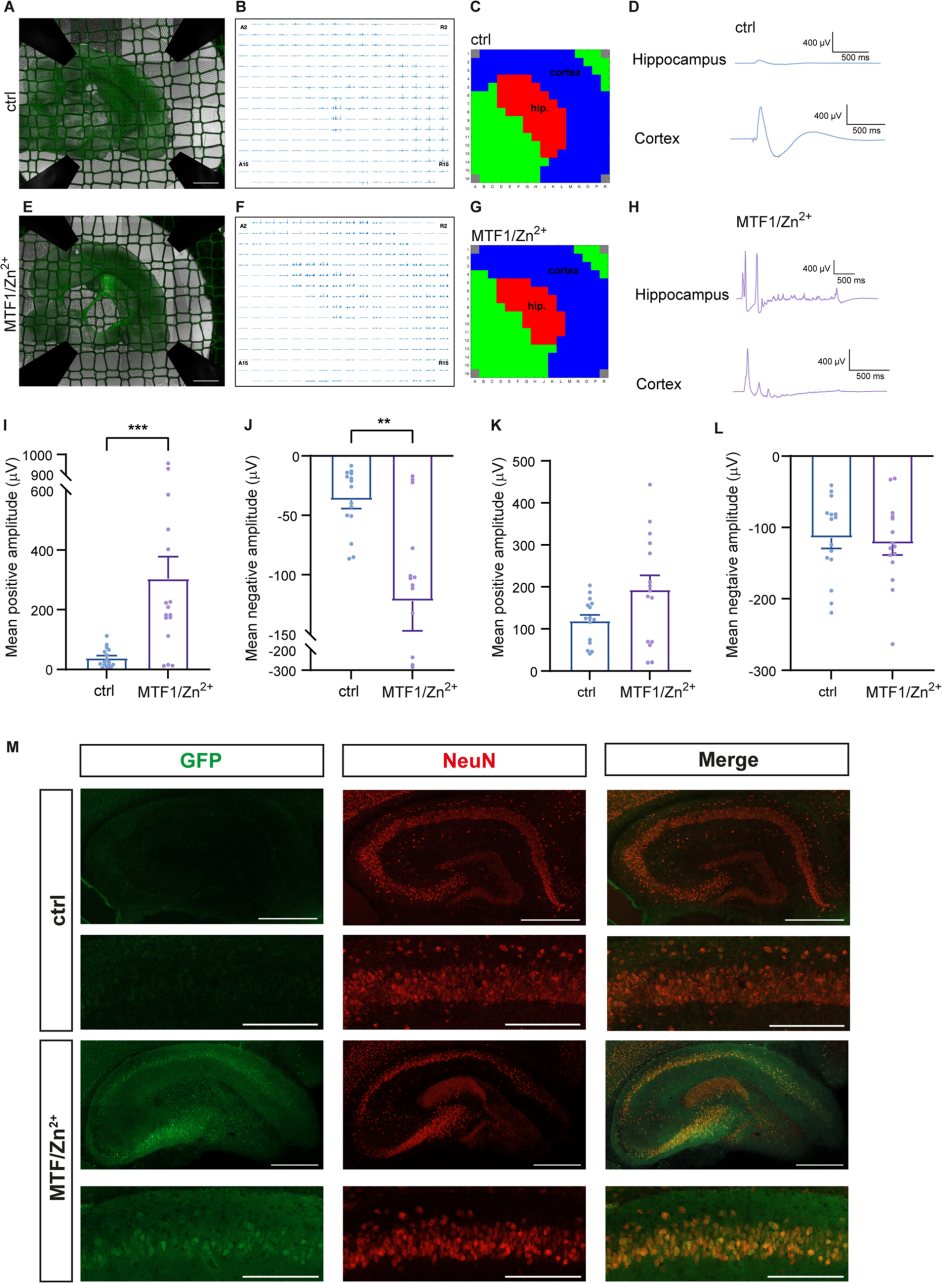
The MTI transcriptional unit is activated by MTF1/Zn^2+^ in the neuronal population in *ex vivo* mouse brain slices. (**A—H**) Low-pass filtered (50 Hz) multi-electrode array (MEA) recordings of mouse organotypic brain slice cultures (OBSCs) transduced with rAAV-MTI-Venus at days in vitro (DIV) 0 under basal conditions (**A-D**) and after AAV-mediated MTF1 expression and Zn^2+^ treatment (transduction of rAAV-hSyn-MTF1-2A-SBFP2 at DIV0, and stimulation with 1 µM ZnCl_2_ at DIV7 for 30 min) (**E–H**), slices were analysed at DIV9. (**A**, **E**) Representative images of OBSCs taken directly after the MEA recordings, and (**B**, **F**) corresponding recorded MEA signals. (**C**, **G**) Layer dictionaries were created to distinguish hippocampal and cortical areas of each slice: blue = cortex, red = hippocampus, and green = other. (**D**, **H**) Exemplary waveforms of local field potentials (LFPs) from the hippocampus and cortex in control (**D**) and AAV-mediated MTF expression and Zn^2^⁺-stimulation (**H**) OBSCs. (**I-L**) Mean positive and negative LFP amplitudes in hippocampal (**I**, **J**) and cortical (**K**, **L**) regions. (Mann–Whitney test, ** = *P* ≤ 0.01, ***P ≤ 0.001; *N* = 5 independent experiments, *n* ≥ 15 replicates per condition). (**M**) Fluorescence-immunolabeling using GFP and NeuN antibodies on OBSCs transduced with rAAV-MTI-Venus (ctrl, upper panels) and after MTF1/Zn^2+^ treatment (MTF1/Zn.^2+^, lower panels) in the hippocampal region. Scale bars: 500 μm (top rows) and 200 μm (bottom rows)

The use of ex vivo organotypic brain slice cultures (OBSCs) allows the analysis of electrophysiological and molecular properties of specific cell populations in brain tissue with preserved anatomy, cytoarchitecture, and synaptic integrity [36]. To test whether the MTI transcriptional unit can also be used to label Zn^2+^/MTF1-responsive neurons in such ex vivo slices, we first determined the circumstances for optimal Zn^2+^/MTF1-stimulation in OBSCs. Slices were transduced at DIV0 with rAAV-MTI-Venus and rAAVs overexpressing the MTF1 protein (rAAV-hSyn-MTF1-2A-SBFP2). One week later, the slices were incubated in various concentrations of Zn^2+^ solution (1 µM, 50 µM, 100 µM and 200 µM ZnCl_2_) for 30 and 60 min (*N* = 2, *n* = 2). Subsequently, the slices were kept in culture for two more days and analysed for their survival rate and Venus expression. Here, Venus fluorescence was used as a visual proxy for neuronal health, as it is expressed in the targeted neuronal population and diminishes when neurons are damaged or lost. A strong reduction in survival rate (visualized by reduced Venus expression) was observed for the slices incubated in 100 and 200 µM ZnCl_2_, whereas slices incubated for 30 min at 1 µM ZnCl_2_ showed the best survival rate (data not shown). Subsequent MEA analysis of OBSCs treated with this Zn^2+^/MTF1-paradigm (DIV0: rAAV-hSyn-MTF1-2A-SBFP2; DIV7: 30 min, 1 µM ZnCl_2_, *N* = 5, *n* = 15) and control OBSCs transduced with rAAV-MTI-Venus but without the Zn^2+^/MTF1-paradigm (*N* = 5, *n* = 15), confirmed the viability of the Zn^2+^/MTF1-stimulated OBSCs (Fig. 3A-H). In addition, differences in the amplitudes of the local field potentials (LFPs), which reflect low-frequency extracellular voltage fluctuations generated by the summed synaptic activity of neuronal populations, were evident in the hippocampal region of OBSCs stimulated with Zn^2+^/MTF1, confirming the functional effect of the Zn^2+^/MTF1-stimulation (Fig. 3I, J). No differences in LFP amplitudes between Zn^2+^/MTF1-stimulated and control OBSCs were observed in the cortex (Fig. 3K, L).

Next, control OBSCs transduced with rAAV-MTI-Venus, and OBSCs stimulated with the Zn^2+^/MTF1-paradigm as described above, were subjected to immunofluorescence analyses with a GFP-targeting antibody to detect Venus expression and a NeuN-directed antibody to label neurons. Control sections (N = 3 independent experiments, *n* = 6 replicates per condition) showed little fluorescent activity of the MTI transcriptional unit (Fig. 3M, upper panels), whereas Zn^2+^/MTF1-treated sections (*N* = 3 independent experiments, *n* = 6 replicates per condition) exhibited strong neuronal expression of the MTI reporter unit (Fig. 3M, lower panels), indicating that the MTI transcriptional unit can be used to label Zn^2+^/MTF1-activated cells in OBSCs.

Finally, we assessed the MTF1-specificity of the MTI reporter construct using a previously described dominant-negative variant of MTF1 (MTFdC) [3]. MTFdC retains the DNA-binding domain and can still bind to MREs in the DNA; however, it lacks the C-terminal transactivation domain and therefore cannot activate transcription.

We first transduced OBSCs with rAAV-MTI-mRuby3 at DIV0. Following Zn^2+^-stimulation at DIV7 (1 µM ZnCl_2_, 30 min), an increase in MTI reporter activity was observed (Suppl. Figure 2A). However, when slices were transduced at DIV0 with the dominant-negative MTF1 variant and subsequently stimulated with Zn^2+^ at DIV7, no increase in MTI-reporter activity was detected (Suppl. Figure 2A). In contrast, overexpression of intact MTF1 confirmed activation of the MRE-MTI-mRuby3 following Zn^2+^/MTF1-stimulation (Suppl. Figure 2B). These results demonstrate the specificity of our reporter construct for MTF1.

### The MTI Transcriptional Unit is Activated Early after Pilocarpine-induced SE

**Fig. 4:**
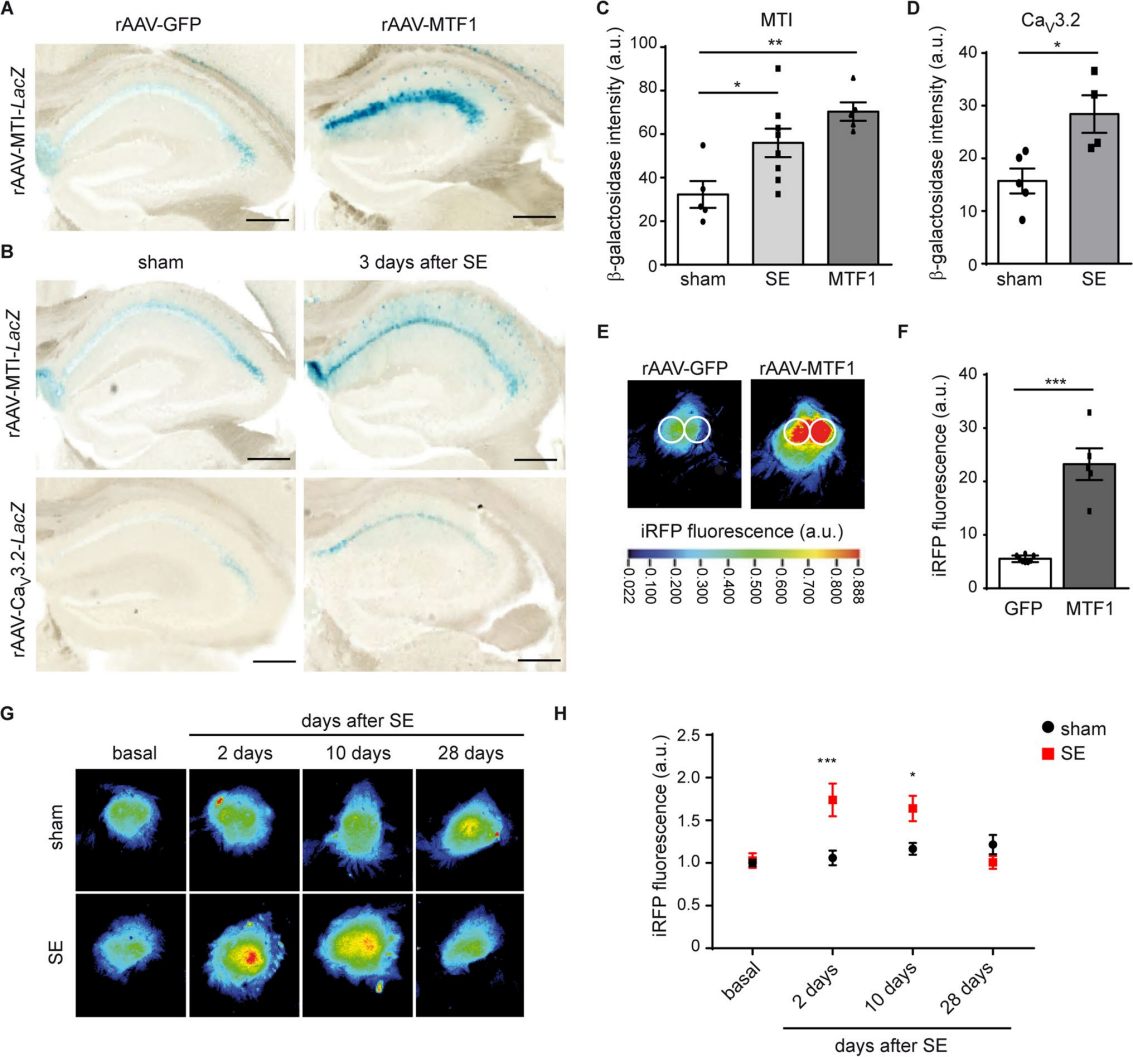
The MTI transcriptional unit in the pilocarpine-induced status epilepticus (SE) model. (**A**) *Lac*Z staining of the MTI transcriptional unit in the hippocampal region under basal conditions (rAAV-GFP) and two weeks after rAAV-hSyn-MTF1-2A-SBFP2 (rAAV-MTF1) transduction. A strong accumulation of *Lac*Z staining was observed in hippocampal CA1 region after rAAV-MTF1 transduction, indicating that the MTI transcriptional unit is sensitive for MTF1 in vivo*.* Scale bars = 200 µm. (**B**) *Lac*Z staining of the MTI transcriptional unit (upper panels) and *Ca*_*V*_*3.2* core promoter (lower panels) in sham and mice three days after pilocarpine-induced SE. Scale bars = 200 µm. (**C**) Quantification of rAAV-MTI-*LacZ* expression of sham -, pilocarpine-induced SE- and MTF1 treated mice. (One-way ANOVA: *P* = 0.0043, *F(2,15)* = 7.987, Tukey’s multiple comparisons test, *P ≤ 0.05, **P ≤ 0.01, *n* = 5 sham, 8 SE, 5 MTF1)*.* (**D**) Quantification of *LacZ* expression under the Ca_v_3.2 core promoter of sham- and pilocarpine-induced SE-injected animals (t-test: *P ≤ 0.05, *n* = 5 sham, 4 SE) (**E**) Near-infrared in vivo imaging. Representative pseudo color visualization of in vivo iRFP signal of a recorded rAAV-MTI-iRFP mouse with exposed skull under basal conditions (rAAV-GFP; left panel) and 14 days after rAAV-MTF1-2A-SBFP2 transduction (rAAV-MTF1; right panel). Regions of interest (ROIs) were defined above the hippocampal region and the surface radiance was defined in arbitrary units (a.u.). The color bar indicates the total fluorescence efficiency. (**F**) Quantification of E (t-test: ***P ≤ 0.001, *n* = 8 GFP, 5 MTF1) (**G**) Representative examples of a recorded rAAV-MTI-iRFP mouse under basal conditions and after pilocarpine-induced SE longitudinally. (**H**) Quantification of iRFP signals of sham- and pilocarpine-induced SE-injected animals (Two-way ANOVA, *Sidak´s multiple comparisons test*, *P ≤ 0.05, ***P ≤ 0.001, *n* = 8 sham, 6 SE)

To probe whether the MTI transcriptional unit also reflects the Zn^2+^/MTF1-activity in vivo, rAAVs carrying the *Lac*Z reporter gene under the control of the MTI transcriptional unit (rAAV-MTI-*Lac*Z, Fig. 2B) were injected into CA1 pyramidal cells (PCs) of adult mice. A strong increase in *Lac*Z staining was observed after co-transduction with MTF1 viral particles (Fig. 4A, C). In addition, three days after pilocarpine-induced SE, strong *Lac*Z accumulation was observed for the MTI transcriptional unit (Fig. 4B; upper panel, Fig. 4C) as well as for the Ca_V_3.2 core promoter (Fig. 4B; lower panel, Fig. 4D), paralleling the “Zn^2+^-MTF1-Ca_V_3.2” cascade of epileptogenesis [3]. To analyze the activation of the MTI transcriptional unit at different time points after pilocarpine-induced SE, we next performed in vivo imaging using rAAVs expressing an infrared fluorescent protein (iRFP^713^) under the control of the MTI transcriptional unit (Fig. 2B) injected in the CA1 hippocampal region of adult mice. iRFP in vivo imaging is a non-invasive, simple, and low cytotoxic technology that has also been used to track tumor tissue growth, metabolites, and gene expression [37]. Enhancement of MTI transcriptional activity was observed after transduction with MTF1 (Fig. 4E, F) and early after pilocarpine-induced SE (Fig. 4G, H). The iRFP signal increased significantly at 2 days (1.7 fold, p ≤ 0.001), decreased slightly but remained significant at 10 days (1.6 fold, *p* ≤ 0.05), and returned to baseline levels 28 days after compared with sham littermates (Fig. 4G, H). These results were consistent with the monitoring of Ca_v_3.2 promoter activity after SE [35] and confirmed the activation of the “Zn^2+^-MTF1-Ca_v_3.2” cascade during epileptogenesis.

### MTI Transcriptional Unit Overlaps with Cells Exhibiting Cacna1h Promoter-driven Venus Reporter Activity

**Fig. 5:**
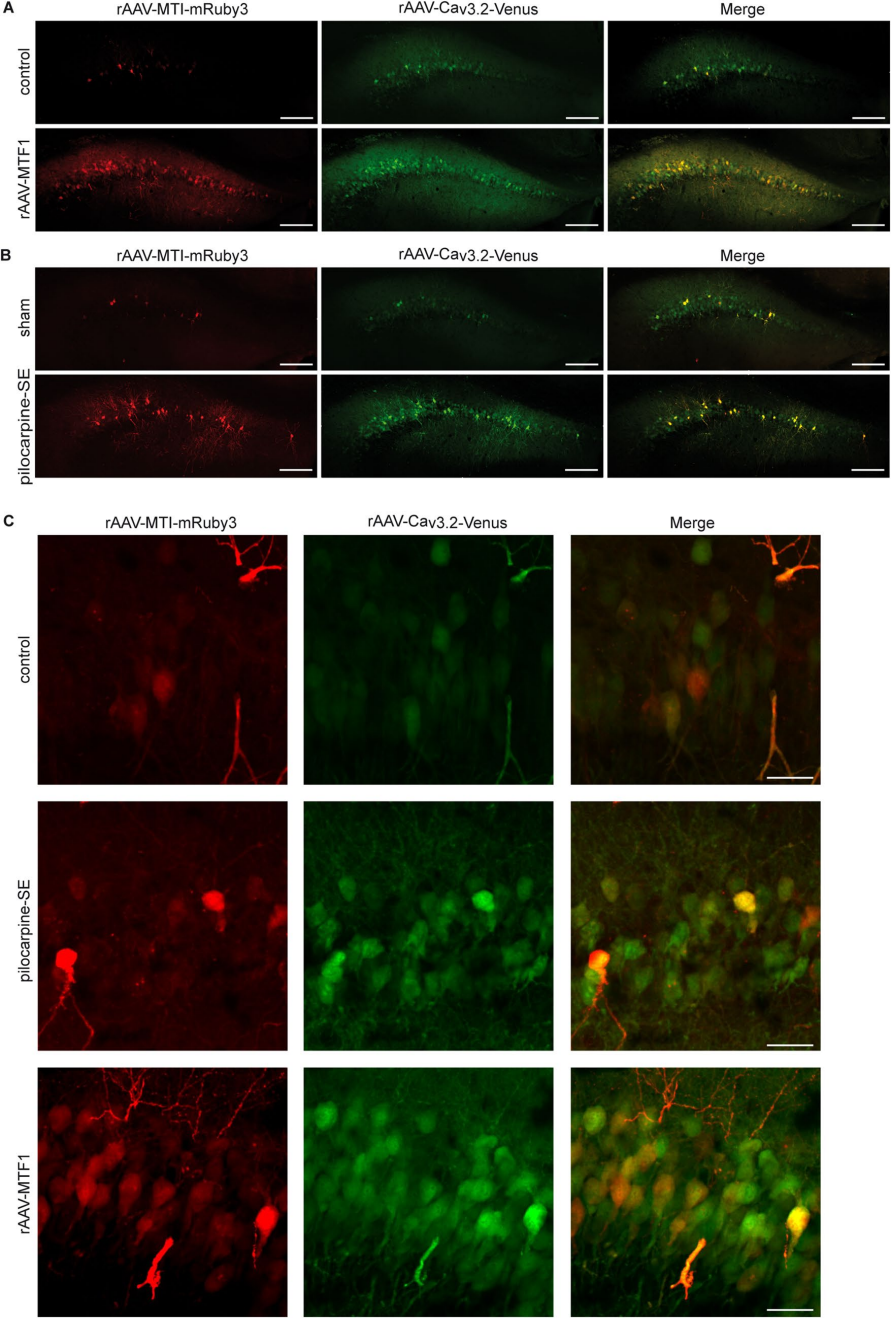
The MTI-transcriptional unit co-localizes with cells expressing a *Cacna1h* promoter-driven Venus reporter. (**A**) Representative image of the hippocampal CA1 region of a rAAV-MTI-mRuby3 and rAAV-Ca_v_3.2-Venus injected animal in basal conditions (control; upper panel, *n* = 4) and 14 days after rAAV-MTF1-2A-SBFP2 transduction (rAAV-MTF1; lower panel, *n* = 3). Scale bar, 200 µm. (**B**) Representative image of rAAV-MTI-mRuby3 and rAAV-Ca_v_3.2-Venus injected animal 3 days after pilocarpine induced status epilepticus (SE). (*n* = 4) Scale bar, 200 µm. (**C**) More detailed images of the groups shown in A, B showing a clear overlap between rAAV-MTI-mRuby3 and rAAV-Ca_v_3.2-Venus. Scale bar, 50 µm

Finally, to confirm that our newly developed transcriptional unit can be used as a marker for Zn^2+^-induced MTF1-responsive neuronal populations, we investigated whether the MTI transcriptional unit and *Cacna1h* promoter activity are present in the same cells. To this end, we injected a combination of rAAV particles carrying a *Cacna1h* promoter-driven Venus reporter (rAAV-Ca_v_3.2-Venus), which has been shown to strongly correlate with endogenous Ca_V_3.2 mRNA levels [3, 31, 35], together with rAAV-MTI-mRuby3 particles into the hippocampal CA1 region of mice. After MTF1 overexpression (co-injection with rAAV-MTF1-2A-SBFP2), an increase in Venus and mRuby3 fluorescence intensity was observed, as well as a strong overlap of Venus- (reflecting *Cacna1h* promoter activity) and mRuby3-positive cells (MTI transcriptional unit) (Fig. 5A, C). To test whether this finding could be reproduced during the early phases of epileptogenesis, mice were subjected to pilocarpine-SE or sham-treatment and sacrificed three days later, the time point of the highest Ca_v_3.2 mRNA level after SE [38]. As expected, a strong increase in the fluorescence intensity of rAAV-Ca_v_3.2-Venus and rAAV-MTI-mRuby3 was observed, with strong overlap of the Venus and mRuby fluorescence signals (Fig. 5B, C), indicating that the MTI transcriptional reporter unit can be used to visualize Zn^2+^/MTF1 responsive neuronal populations.

## Discussion

The transcription factor MTF1 is well known for its role in heavy metal homeostasis and oxidative stress. Other studies have shown that MTF1 is involved in a broader range of biological processes, including inflammation (by regulating both pro- and anti-inflammatory cytokines), and neuronal plasticity, through the regulation of β-synuclein expression [39–41]. Furthermore, MTF1 has been reported to have biological significance in several cancer subtypes and has been proposed as a novel prognostic biomarker in low-grade glioma [42, 43]. MTF1 has also been shown to activate Ca_V_3.2 expression in the early phase of epileptogenesis [3] and in social stress-induced anxiety [9]. However, while tools exist to detect cellular MTF1 expression, no method is currently available to visualize Zn^2+^-activated MTF1 molecules in vivo*.* In this study, we developed a genetic tool to label and characterize Zn^2+^/MTF1-activated cellular populations and further investigate them with respect to the expression of Zn^2+^/MTF1 downstream target genes.

Among the five reporter units tested, the construct containing the mouse MTI promoter showed the highest responsiveness to Zn^2+^/MTF1 activation. In primary neurons, we detected MTF-dependent activation of this construct as early as 2 h after Zn^2+^-exposure, consistent with MTF’s established role in rapid cellular reactivity to changes in metal ion concentrations [4]. While application of Zn^2+^ alone resulted in a modest increase in reporter activity, co-application of Zn^2+^ and MTF1 led to a markedly stronger transcriptional response. Since both intracellular Zn^2+^ levels and MTF1 expression are substantially elevated under pathological conditions – such as following SE [3]– we chose to focus on this combinatorial Zn^2+^/MTF1 activation paradigm. Our *ex vivo* and *in vivo* data confirm that the MTI transcriptional unit provides a reliable genetic tool for labeling MTF1 in a Zn^2+^-dependent manner.

**Fig. 6:**
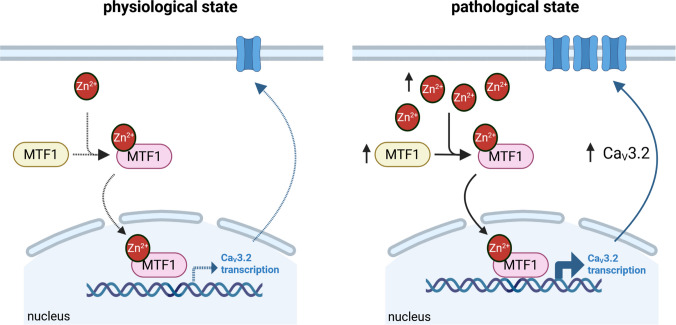
Schematic representation of the ‘Zn^2+^-MTF1-Ca_v_3.2 cascade’ under physiological (left panel) and pathological (right panel) circumstances. After an insult, such as status epilepticus, an increase of intracellular Zn^2+^ and expression of the Zn^2+^-responsive transcription factor MTF1 is observed, resulting in transcriptional activation of the Ca_V_3.2 promoter. Created with BioRender.com

Our *in vivo* data showed that cells activated by Zn^2+^/MTF1 strongly overlap with cells exhibiting *Cacna1h* promoter-driven reporter expression, suggesting a close association with Cav3.2 expression under both physiological and pathophysiological conditions. In addition, our *ex vivo* data showed a stronger increase in network activity measured by the higher LFP amplitudes of Zn^2+^/MTF1-stimulated slices in the hippocampal region compared to the cortical area. As the hippocampus is one of the regions with the strongest Cav3.2 expression, and also shows differential expression of Cav3.2 after pilocarpine-induced SE [38, 44], our data confirm the ‘Zn^2+^-MTF1-Ca_v_3.2 cascade’ and point in the direction that Ca_V_3.2 is a direct target of the Zn^2+^/MTF1-induced promoter activity (Fig. 6). Importantly, while the existence of this cascade was previously hypothesized based on separate lines of evidence, we now show for the first time that activated Zn^2+^/MTF1 and *Cacna1h* promoter activity (as reflected by the Venus reporter) are co-expressed within the same cells, thereby strengthening the cell-intrinsic mechanistic basis for this transcriptional control pathway.

In a wider perspective, this cascade provides a plausible link between metabolic stress and neuronal hyperexcitability during epileptogenesis. Elevated intracellular Zn^2+^ – released synaptically or mobilized from metallothioneins – activates MTF1, which in turn upregulates genes including Ca_V_3.2. Increased Cav3.2 enhances burst firing and lowers the threshold for seizure generation [38, 45]. Thus, the Zn^2+^-MTF1 pathway may function as a molecular amplifier, translating early stress signals into presistent changes in ion channel expression that enhance network excitability and increase seizure susceptibility. This places MTF1 not only as a stress sensor, but as an active contributor to neuronal hyperexcitability and thus epileptogenic remodeling.

MTF1 has been described as a pathogenetically relevant factor in the development of TLE [3, 46]. Because the efficacy of therapeutic approaches is highly dependent on target validation as well as the timing of intervention, a detailed understanding of the cellular behavior of the Zn^2+^/MTF1 signaling cascade would be a prerequisite for the development of therapeutic approaches specifically targeting this pathway. By measuring the iRFP signal under the control of the MTI transcriptional unit in AAV-intracranially transduced sham and pilocarpine-induced SE mice, we observed that MTF1-dependent changes in gene expression occur early after an initial precipitating event. This finding is important considering that the transcriptional regulation of activity-dependent genes could be useful as potential biomarkers for pathological diagnosis and for the validation of antiepileptogenic interventions [47]. Because hippocampal MTF1 mRNA is upregulated 12 h after pilocarpine-SE [3] and MTF1-dependent gene regulation increases immediately after pilocarpine-SE, MTF1 or one of its target genes could thus be used as potential biomarkers. Our present data indicate that neurons with strong *Cacna1h* promoter-driven reporter activity are also Zn^2+^/MTF1-responsive, whereas the absence of detectable MTI reporter labelling in neurons with weaker reporter activity may reflect either biological differences or limitations in fluorescence detection. These data highlight the Zn^2+^/MTF1-cascade as a potential molecular biomarker of overt epileptogenesis as well as a valuable target for anti-epileptogenic therapy strategies.

While our findings provide valuable insights into Zn^2+^/MTF1 signaling during epileptogenesis, it is essential to acknowledge certain limitations inherent to our study. First, although we have established a tool for visualizing Zn^2+^-activated MTF1 signaling, questions may remain regarding its absolute specificity. While we cannot entirely exclude the possibility that other metal ions or signaling pathways influence our system, we experimentally confirmed MTF1-specific activation *ex vivo* using a dominant-negative MTF1 construct. Here, Zn^2+^ stimulation failed to activate the reporter in the presence of MTFdC, supporting that transcriptional activation is dependent on functional MTF1. Nevertheless, additional *in vivo* validation under varying physiological conditions would further strengthen these findings. Second, although we here focused on examining changes within the hippocampus, it remains unclear whether similar mechanisms operate across different brain regions. Finally, the use of AAVs for reporter delivery introduces variability in transduction efficiency across animals and brain regions, potentially affecting the interpretation of spatial and quantitative patterns of reporter expression. Although we used consistent AAV-transduction protocols, subtle differences in viral spread may contribute to inter-sample variability.

In conclusion, despite these limitations, our present data demonstrate that our newly developed Zn^2+^/MTF1-genetic tool enables visualization and investigation of the consequences of intracellular Zn^2+^ elevation under both physiological and pathological conditions, including the perspective of identifying Zn^2+^/MTF1 cascade-controlled gene sets more comprehensively in the future.

## Supplementary Information

Below is the link to the electronic supplementary material.

Supplementary file1 (PDF 1814 KB)

## Data Availability

No datasets were generated or analysed during the current study.
