# Supplementary material for: A New Toolbox to Label Zinc-MTF1 Responsive Neuronal Populations Unravels Cellular Congruence between MTF1 Responses and T-type Calcium Channelopathies in an Experimental Model of Epilepsy
Source: Mol Neurobiol. 2025 Dec 13;63(1):288. doi: 10.1007/s12035-025-05433-z (PMC12701871; doi:10.1007/s12035-025-05433-z)
Supplement: Supplementary file 1 — Supplementary file1 (PDF 1814 KB) [file 12035_2025_5433_MOESM1_ESM.pdf]

Supplementary Information

A new toolbox to label zinc-MTF1 responsive neuronal populations unravels cellular congruence between MTF1 responses and T-type calcium channelopathies in an experimental model of epilepsy

Annachiara Meconi[1], Katharina Schmied[2], Aniella Bak[2], Julika Pitsch[3], Henner Koch[2], Susanne Schoch[1], Albert J. Becker[1,§] and Karen M.J. van Loo[2,4§*]

[1]Institute of Cellular Neurosciences II (IZN II), Medical Faculty, University of Bonn, Bonn, Germany
[2]Department of Epileptology, Neurology, RWTH Aachen University, Aachen, Germany
[3]Department of Epileptology, University Hospital Bonn, Bonn, Germany
[4]Department of Neurosurgery, RWTH Aachen University, Aachen, Germany
§ equally contributed

Supplementary Figure 1

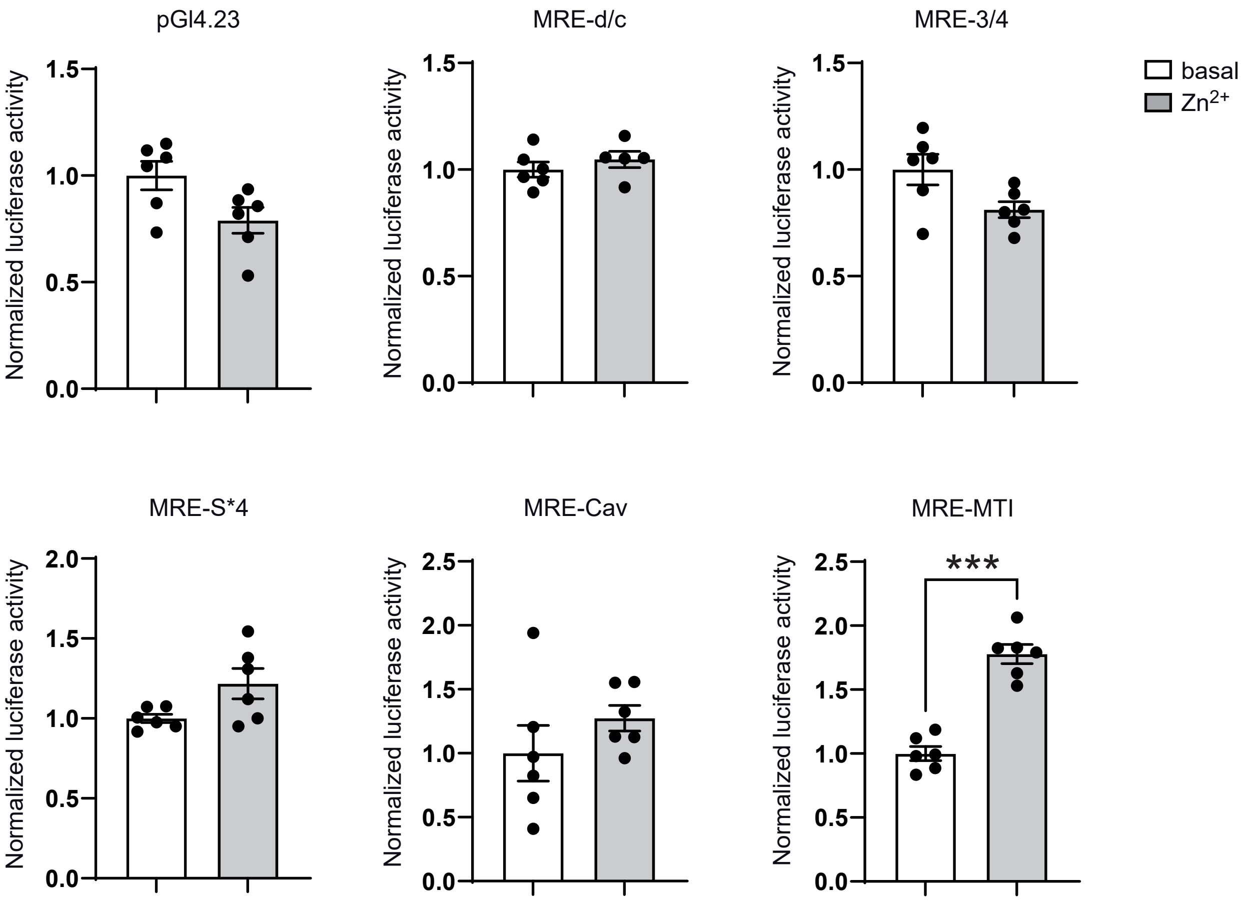

Suppl. Fig. 1 Increases in intracellular Zn^{2+} activate only the MRE-MTI transcriptional unit in NG108-15 cells. Luciferase activity of the minimal promoter fragment (pGl4.23) and five different transcriptional units under control conditions (basal) and after Zn^{2+}-stimulation (200 µM Zn^{2+} and 50 mM KCl for 4 h). A significant increase in luciferase activity was only observed for MRE-MTI (1.78-fold). Student's *t*-test with Welch's correction; ***$P \leq 0.001$; N = 2 independent experiments, n = 3 replicates per condition).

Supplementary Figure 2

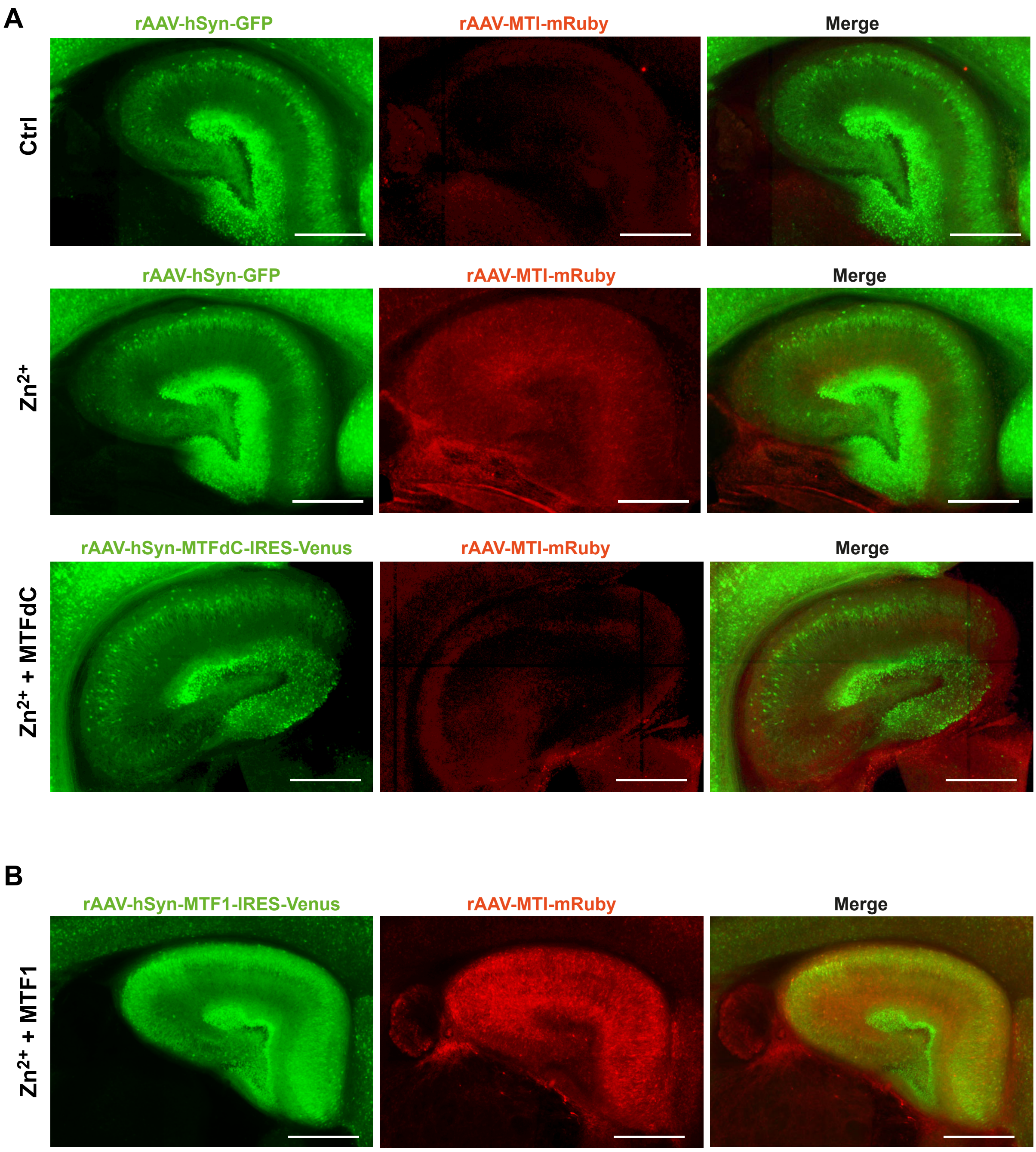

Suppl. Fig. 2 MTF1-specific activation of the MRE-MTI reporter in *ex vivo* mouse brain slices. (A) Representative fluorescent images of organotypic brain slice cultures (OBSCs) transduced with rAAV-MTI-mRuby and co-transduced with rAAV-hSyn-GFP (upper two panels) or rAAV-hSyn-MTFdC-IRES-Venus (lower panel). Upon stimulation with Zn^{2+} the increase in MTI reporter activity (middle panel) was blocked when using the MTFdC variant (lower panel). Scale bars: 500 µm (B) A strong increase in MTI reporter activity was observed after AAV-mediated MTF1 application and subsequent Zn^{2+} treatment. Scale bars: 500 µm.
